# Thermal Behavior of Sweet Potato Starch by Non-Isothermal Thermogravimetric Analysis

**DOI:** 10.3390/ma12050699

**Published:** 2019-02-27

**Authors:** Ying Liu, Liutao Yang, Chunping Ma, Yingzhe Zhang

**Affiliations:** 1 College of Chemical Engineering, Guizhou Institute of Technology, Guiyang, Guizhou 550003, China; liuychemistry@163.com; 2 Key Laboratory of Light Metal Materials Processing of Guizhou Province, Guizhou Institute of Technology, Guiyang, Guizhou 550003, China; machunping81@163.com (C.M.); zhangyzhehunan@aliyun.com (Y.Z.); 3 College of Materials and Metallurgical Engineering, Guizhou Institute of Technology, Guiyang, Guizhou 550003, China

**Keywords:** starch, kinetic analysis, thermal degradation, activation energy, mechanism

## Abstract

In this study, X-ray diffraction (XRD), thermogravimetric analysis (TGA), and differential scanning calorimetry (DSC) methods were used to study the structure, the thermal degradation kinetics, and the thermogram of sweet potato starch, respectively. The thermal decomposition kinetics of sweet potato starch was examined within different heating rates in a nitrogen atmosphere. Different models of kinetic analysis were used to calculate the activation energies using the thermogravimetric data of the thermal degradation process. The activation energies got from Kissinger, Flynn–Wall–Ozawa, and Šatava–Šesták models were 173.85, 174.87, and 174.34 kJ·mol^−1^, respectively. Thermogravimetry–Fourier transform infrared spectroscopy (TG-FTIR) analysis showed that the main pyrolysis products included water, carbon dioxide, and methane.

## 1. Introduction

Starch is one of the most widely investigated biopolymers made from rice, potato, and maize. It is an unusual material with a more complicated structure than that of synthetic polymers. This polymer is a mixture of amylopectin, highly branched polysaccharides with α-1,4 backbone chains linked by α-1,6 bonds, and amylose, a dominant linear structure of α-1,4-linked glucose units [1]. 

Starch decomposes when heated, and the degradation of starch is a complicated process. In practical applications, it may be enough to consider the basic characteristics of the thermal decomposition process using some simple mechanisms. From the point of view of science and industry, thermal degradation and stability is a very important problem. The heat treatment of starch has been widely used in the food industry and in starch-based materials [2,3]. 

Thermal analysis is a group of techniques for the rapid assessment of thermal stability, decomposition parameters, adsorbed water content, crystal water content, and the thermal degradation dynamics [4]. Thermogravimetric (TG) and differential scanning calorimetry (DSC) methods have been used as prevalent techniques to evaluate the thermal behavior of polymers. The assessment of thermal stability and degradation dynamics of the starch is very important in practical applications. Non-isothermal tests are mostly preferred for the dynamic analysis. To this end, many models, such as Kissinger, Flynn–Wall–Ozawa, Šatava–Šesták, and Friedman, were established to study the dynamic parameters of various materials based on the Arrhenius equation [5,6,7,8,9]. 

There have been some reports about the degradation of raw or processed starches from many different sources using different thermal analysis models [1,10]. Dewatering and pyrolysis were usually deemed as the two main stages related to the decomposition mechanism of starch. The distinctive microstructure of starch and its multiple phase transformation in the process of heating give an excellent approach to explain the structure–processing–performance relationship. The achievements in the field can increase the knowledge of polymer materials, in particular, that of natural polymers. Thermogravimetric analysis was used to investigate the pyrolysis of corn starches with different amylose/amylopectin ratios in nitrogen [11]. The result showed that the decomposition of starches with a higher amount of amylopectin needed more activation energy. The effect of chemical modification like acetylation, hydroxy propylation, and benzylation on the thermal property of yellow sorghum starch was investigated [12]. DSC tests showed that the gelatinization temperature of modified yellow sorghum starches was lower than that of natural starch. Thermogravimetric analysis was used to study the thermal behavior of mixtures of rice bran and high-density polyethylene under a nitrogen atmosphere, and the results were compared with those of individual materials [2]. The kinetics of thermal degradation of potato starch was investigated only using Flynn–Wall–Ozawa method [13], and this result was not systematic. The thermal stabilities of purple sweet potato anthocyanins were studied with varying concentrations of ascorbic acid, but this study was performed at a low temperature (70–90 °C) [14].

The main purpose of this study was to investigate the thermal behavior of sweet potato starch using TG and DSC. In this study, three methods were used: XRD to study its structure, TG to investigate the thermal degradation processing, and DSC to survey the thermogram of sweet potato starch. The thermal degradation data were used to analyze the thermal degradation kinetics with different isoconversional models like Kissinger, Šatava–Šesták, and Flynn–Wall–Ozawa models, which were used to evaluate the activation energy of sweet potato starch and its activation energy of thermal degradation. The decomposition activation energies obtained in this study can help to evaluate the thermal stability of sweet potato starch used in the food and chemical processing industry.

## 2. Materials and Methods 

### 2.1. Materials

The sweet potato starches used in this study were purchased from Guiyang Jincheng Co., Ltd. (Guizhou, China), and the moisture content is less than 1%. The dried samples were crushed and sieved using a sieve of 0.15 mm in order to get uniform particles for TG/DSC measurements. The samples were kept in airtight packages.

### 2.2. Methods

#### 2.2.1. X-ray Diffraction

The X-ray diffraction was measured using a diffractometer (Siemens D5000, Bensheim, Germany) instrument, which was operated at 40 kV and 40 mA, and 2*θ* range from 5° to 72° with a step size or sampling interval of 0.02°. From the XRD patterns, the interplanar spacings (*d*) were calculated by Bragg’s equation. In order to get the crystallization fraction of sweet potato starch, the software MDI Jade 5.0 was used.

#### 2.2.2. TG/DSC Analysis

A thermal analyzer (STA-625, Reometric Scientific, Rigaku, Tokyo, Japan) was used for obtaining TG and DSC curves. The thermal analyses were carried out in alumina crucibles under dry a argon atmosphere with a flow rate of 10 mL·min^−1^. The starting temperature was 35 °C. The rates of heating were 5, 10, 15, and 20 °C·min^−1^, respectively. Each sample was about 5 mg.

#### 2.2.3. Thermogravimetry-Fourier Transform Infrared Spectroscopy (TG-FTIR) Test

Thermogravimetry–Fourier transform infrared spectroscopy (TG-FTIR) analysis was obtained by TG 209F3/Tensor 27 (Netzsch/Bruker, Bavaria, Germany), heating under N_2_ from room temperature to 600 °C with a heating rate of 20 °C·min^−1^.

#### 2.2.4. Crystallinity Index (X_c_)

The crystallinity index is an important parameter, which has an important effect on the utilization of materials. X-ray diffraction can provide a qualitative or semi-quantitative assessment of the crystallinity index. The value of the crystallinity index (X_c_) was obtained using the following formula:

X_c_ = H_c_/(H_c_ + H_a_),

where H_c_ and H_a_ are the intensities of the crystalline and amorphous parts, respectively.

### 2.3. Theoretical Background

The thermal degradation speed dα/dt was the function of T and α, and the formula is shown in Equation (1) [15].

dα/dt = k(T) f(α),(1)

where T is degradation temperature, α is conversion rate, f(α) is the conversion function based on the mechanism of reaction, and k is the rate constant related to T, which depends on the Arrhenius equation:

K = A exp(−E/RT),(2)

where A is the pre-exponential factor, E is activation energy, and R is the gas constant.

The following expression is obtained in conjunction with Equations (1) and (2):

dα/dt = A exp(−E/RT) f(α),(3)

where β is the heating rate, which was a constant. Meanwhile, dα/dt was expressed by the following function:

dα/dt = (dα/dT) (dT/dt) = β(dα/dT).(4)

The following equality was obtained in conjunction with Equations (3) and (4):

dα/dT = (A/β) exp(−E/RT) f(α).(5)

Equation (5) is a general non-isothermal kinetic function.

#### 2.3.1. The Kissinger Technique

The Kissinger technique was a differential approach for studying the thermal decomposition process. The activation energy Ea was calculated with the following formula based on Kissinger [16].

Y[ln(β/T_m_^2^)] = {ln(AR/E) + ln[n (1 − α_m_)]^n − 1^} − Ea/R × X(1/T_m_), (6)

where α_m_ and T_m_ are the fastest conversion ratio and the corresponding temperature, respectively and (dα/dt)_m_ and n(1 − α_max_)^n−1^ were approximately 1. The Ea value was obtained from the slope of a line of ln(β/(T_m_^2^))vs.(1000/T_m_).The intercept of the line was expressed with the following formula:

I = ln (AR/E)(7)

The lnA was obtained from the I value.

#### 2.3.2. Flynn–Wall–Ozawa Technique

The kinetic technique was derived from Flynn, Wall, and Ozawa methods based on the Arrhenius equation, which was expressed with the following formula [17]:

Y(lgβ) = {lg[AE/RG(α)] − 2.315} − 0.4567Ea/R × X(1/T_m_). (8)

The Ea value was obtained from the slope of the line of lgβ vs. (1000/T_m_).

#### 2.3.3. Šatava–Šesták Technique

The Šatava–Šesták method is only suitable for research on solid phase non-fixed-temperature thermal decomposition dynamics [18]. Due to mathematical strict illation, the result obtained from this method is quite reasonable. The Šatava–Šesták technique equation is as follows:

Y[lgG(α)] = {lg[AsEs/Rβ] − 2.315} − 0.4567Es/R × X(1/T_m_),

where Es is the apparent activation energy, As is the pre-exponential factor, and G(α) is the integral mechanism function.

For a fixed heating rate β_i_, the corresponding T and α were substituted into the equation, and the equation obtained is as follows:

Y[lgG(α)] = {lg[AsEs/Rβ_i_] − 2.315} − 0.4567Es/R × X(1/T_m_).

Es was obtained according to the slope. Different kinetic models should be complementary and not competitive.

## 3. Results

### 3.1. Structural Analysis of the Potato Starch

**Figure 1:**
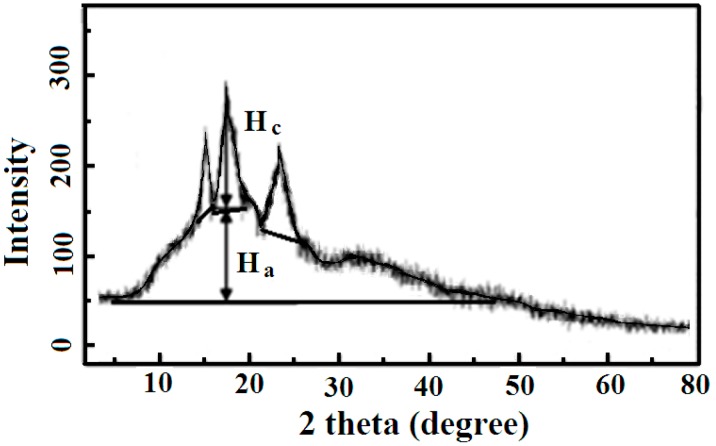
XRD patterns of the sweet potato starch sample. H_a_ and H_c_ are the amorphous and crystalline profiles, respectively.

The sweet potato starch is mainly composed of amylose and amylopectin whose crystal structure was researched by XRD. As shown in Figure 1, the XRD profile of sweet potato starch, the sweet potato starch exhibits three strong peaks at 15.12°, 17.12°, and 23.45°, and three weak peaks at 11.33°, 20.13°, and 26.51°. The crystalline fraction [19] of sweet potato starch was 19%, which is lower than that of banana, cassava, and corn starch [1]. The low crystallinity of sweet potato starch could be ascribed to the absence of a crystalline amylopectin phase. The contents of amylose and amylopectin were 20.7% and 79.3%, respectively, according to the method reported in the literature [20]. 

The crystallinity index (X_c_) of sweet potato starch is 0.56. Since the crystallinity depends mainly on the crystallization of amylopectin, the X_c_ value of sweet potato starch is positively related to the content of amylopectin.

### 3.2. Thermal Degradation

**Figure 2:**
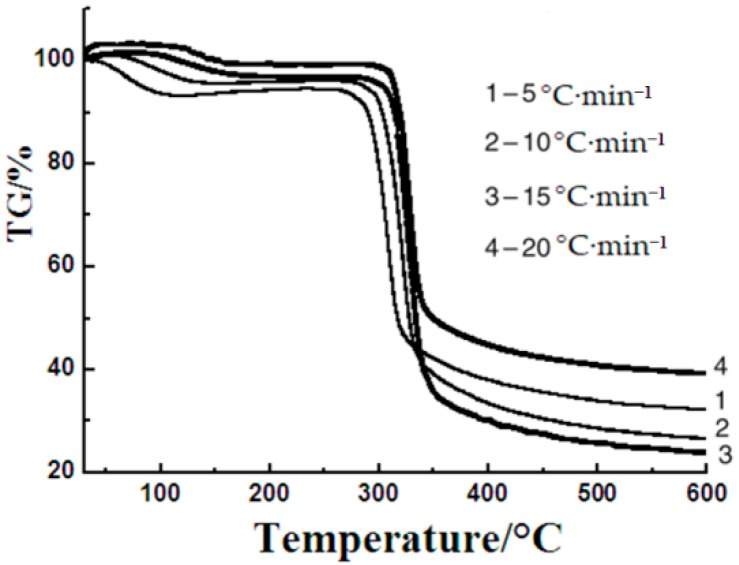
The thermogravimetric (TG) curves of sweet potato starch at different heating rates.

Thermal degradation is a very important part of thermal procedure. Thermogravimetric analysis is a very common method used for researching the thermal degradation process. The thermogravimetric curves of sweet potato starch were measured with different heating rates to study the thermal degradation process with different non-isothermal techniques. Figure 2 shows the thermogravimetric curves of sweet potato starch at different heating rates. These curves present three primary mass loss parts. The initial temperature of each part was identified as the critical point in the TG curves. The initial stage is the desiccation, which starts instantly when the temperature just rises and ends at about 120 °C. The percentage of mass loss in this part depends on the moisture content of the starch samples. The second stage is the main degradation stage, which finishes at around 350 °C. Pyrolysis of starches in this step has been reported to release water, carbon dioxide, carbon monoxide, acetaldehyde, furan, and 2-methyl furan [11]. Thermal decomposition has usually been regarded as the important process associated with the degradation mechanisms of starches. The degradation of amylose and amylopectin happened in this step. The last step ends with the formation of carbon black between 350 and 600 °C. The foremost degradation temperatures were 60, 65, 76, and 80 °C at heating rates of 5, 10, 15, and 20 °C·min^−1^, respectively. The TG data of sweet potato starch in Table 1 was obtained from Figure 2.

The dehydration process is generally not considered to affect the thermal decomposition of starch, because all water will evaporate before the decomposition of the sample in the open system. Even though the form of the thermogravimetric curves does not change, the starting temperature of decomposition is the same, and the maximum of temperature increases with the increasing heating rate. This phenomenon is probably ascribed to a heat transfer problem between the sample and the equipment [21], and the reason may also be that the rapid heating leads the sample to the given temperature rapidly as a result of the increased thermal lag [22,23]. These curves were used for the calculation of dynamics parameters including activation energy and pre-exponential factor.

### 3.3. Kinetics of Thermal Decomposition Analysis

In order to get the dynamics parameters and the most probable mechanism of the thermal degradation process, the thermogravimetric curves at different heating rates were dealt with using three kinetic models.

#### 3.3.1. The Kissinger Model

**Figure 3:**
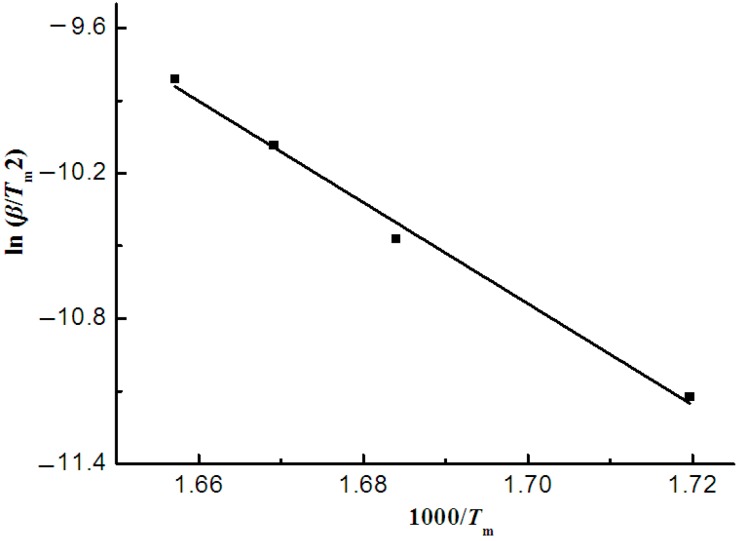
Kissinger plots of potato starch.

The Kissinger model was used to analyze the thermogravimetric curves of sweet potato starch. Figure 3 depicts the fitted curve of ln(β/T_m_^2^) vs. 1000/T_m_, where slopes give −E/R, and the correlation coefficient (R^2^) is 0.9930. Thus, the Kissinger model was appropriately applied in sweet potato starch. The Kissinger model was used to roughly calculate the activation energy of the thermal degradation using the peak value of the thermogravimetric curves. The activation energy and pre-exponential factor got from the line were 173.85 kJ·mol^−1^ and 27.85 min^−1^, respectively. In general, the activation energy value was in the maximum weight loss rate or in the largest heat absorption [24]. 

#### 3.3.2. Flynn–Wall–Ozawa Model

**Figure 4:**
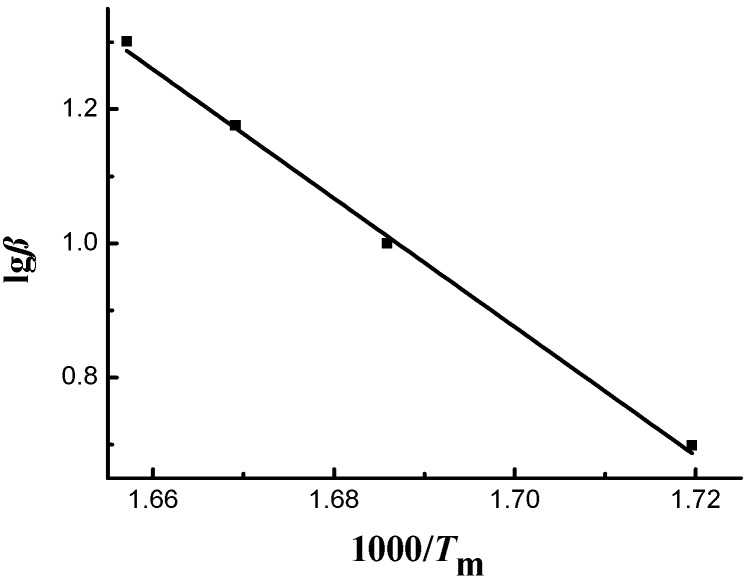
Plots of sweet potato starch.

The Flynn–Wall–Ozawa model was also employed to process the thermogravimetric curves of sweet potato starch and calculate the corresponding dynamics parameters. From Figure 1, the peak temperatures of thermogravimetric curves at different heating rates, T_m_, were obtained. Figure 4 illustrates the linear plot of lgβ vs. 1000/T_m_, where slopes give −E/R. The linear correlation coefficient was 0.9981, and the activation energy was 174.87 kJ·mol^−1^. Thus, the Flynn–Wall–Ozawa model can also be applied to sweet potato starch.

#### 3.3.3. Šatava–Šesták Model

**Figure 5:**
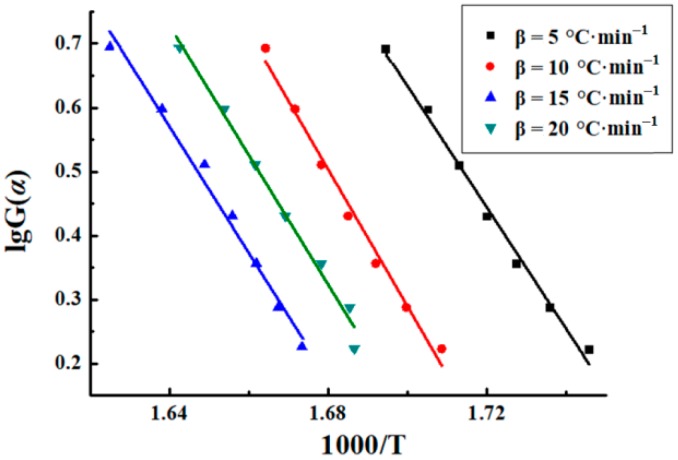
Plots of sweet potato starch.

The Šatava–Šesták model was also used to process the thermogravimetric curves of sweet potato starch. The mechanism function G(α) = −ln(1 − α) was ultimately chosen after comparison with the others. Figure 5 depicts the linear plot of lgβ vs. 1000/T_m_, where slopes give −0.4567E/R. The fitting curves were approximately parallel, and the mean correlation coefficient (R^2^) of the fitted curves was −0.9935. Activation energy is usually regarded as the energy barrier to control the bond breaking or bond reapportioning step [25]. The activation energies got from the slope (−0.4567E/R) are shown in Table 2, and the average value is 174.34 kJ·mol^−1^.

The activation energies obtained by the three models are similar, and the Student’s t-test analysis showed that the three values belong to this same population. The high consistency of the values confirmed the reliability of the calculation and also confirmed the predictive ability of dynamics theory [26]. Therefore, the values of activation energies were reasonable. In fact, different kinetic models should be complementary and not competitive [27]. The activation energy alone is insufficient to forecast and simulate the entire process of thermal degradation. A satisfactory degradation model should at least include a set of activation energy and pre-exponential factor. However, since the activation energy can provide important information about the transition energy required to initiate a reaction, in this study, the range of activation energies can help to investigate the thermal stability of sweet potato starch.

### 3.4. DSC Studies

**Figure 6:**
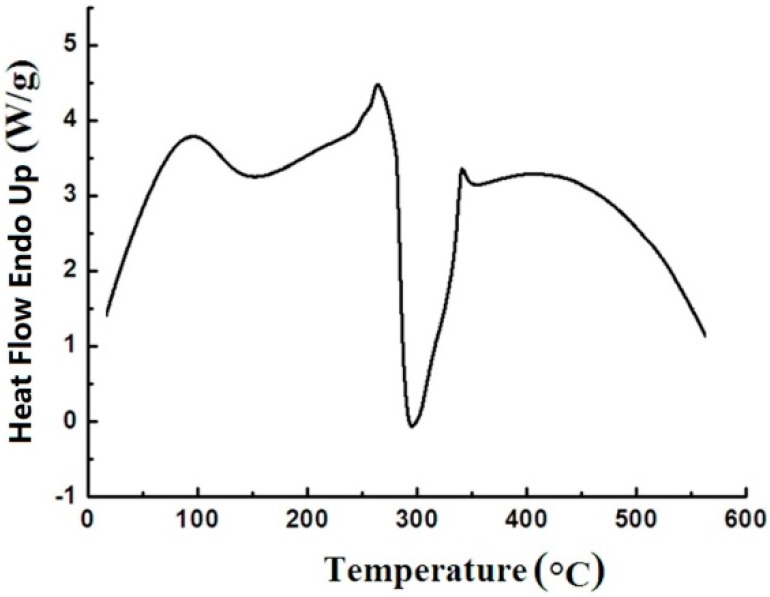
Curve of sweet potato starch under a heating rate of 15 °C min^−1^.

The DSC curve was used to study the thermal transformation occurring in the process of heating under an inert atmosphere. The typical DSC curve of sweet potato starch in a range of 40–580 °C is presented in Figure 6. The peaks at a low temperature (95 °C) are endothermic peaks of gelatinization. Just before this exotherm, there is an endotherm at 270 °C. After opening the DSC pans, the starch sample was yellow to brown. This change is believed to be due to the interruption of long chains. A large exotherm at about 280 °C nearly covers all the thermogram transition which represents the decomposition of starch. The result coincided with those obtained previously from thermogravimetric curves. After opening the DSC pans, the starch sample was carbonized when the temperature was greater than 280 °C. The carbon black directly indicated that the chains of macromolecules and the rings of low molecular glucose had been destroyed.

### 3.5. TG-FTIR Analysis

**Figure 7:**
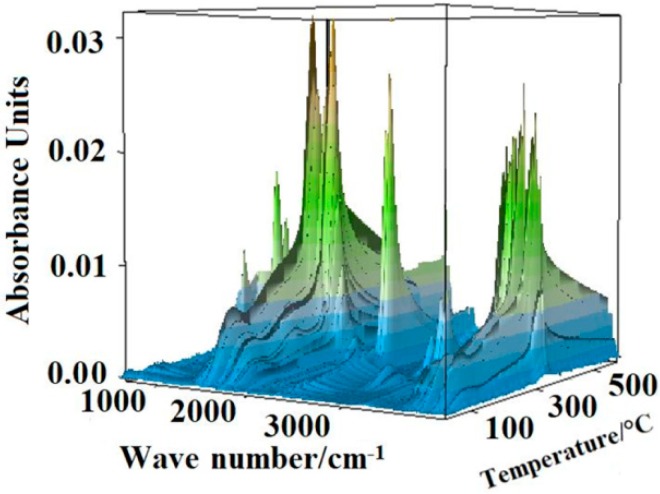
Fourier transform infrared spectroscopy (FTIR) diagram of gaseous products from the pyrolysis of sweet potato starch.

**Figure 8:**
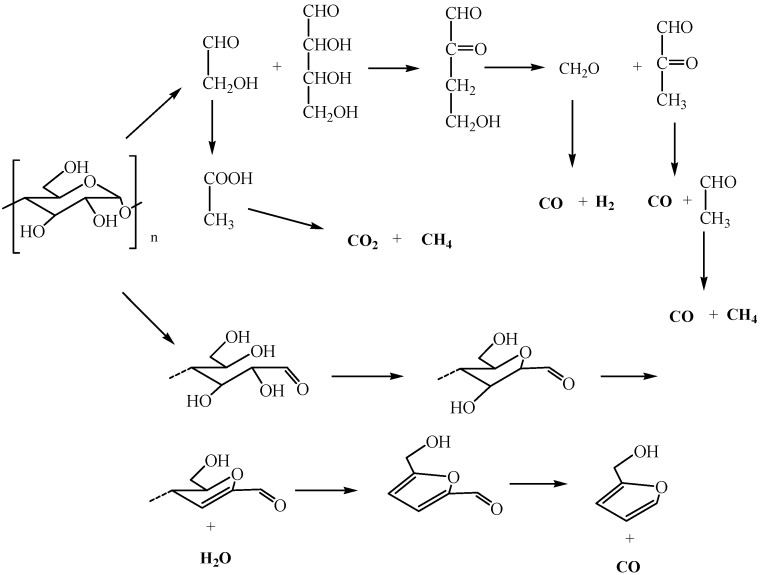
Speculative chemical pathways for the conversion of sweet potato starch.

The three-dimensional (3D) FTIR diagram of gaseous products from the pyrolysis of sweet potato starch is shown in Figure 7. The major products were identified by characteristic absorption bands of a certain compound. Carbon dioxide was released quite strongly, causing the absorption at 2350 and 667 cm^−1^. Mainly water vapor was found, which was characterized by peaks at 3736 cm^−1^. The band at about 2150 cm^−1^ indicated the existence of carbon monoxide. The narrow band at 3014 cm^−1^ was the significant peak of methane. Some other gases were released at the same time, which led to more difficultly in finding the peak of gases with small quantity. The speculative chemical pathway for the conversion of sweet potato starch is indicated in Figure 8 [5]. Under the nitrogen atmosphere, the thermal degradation products of starch mainly included water, carbon dioxide, carbon monoxide, and methane. When the temperature began to rise, water was released and reached its highest value at around 320 °C. At this temperature, the thermal degradation of starch was the fastest, while carbon dioxide and carbon monoxide were released in large quantities. With the increase of temperature, the content of carbon monoxide increased, and methane was released, with a maximum value at about 550 °C.

## 4. Conclusions

X-ray diffraction, thermogravimetric analysis (TGA) and differential scanning calorimetry (DSC) methods were applied for studying the main characteristics of sweet potato starch. The crystallinity index of sweet potato starch is 0.56. Thermogravimetric curves were used to study the thermal degradation kinetics of sweet potato starch by a non-isothermal process. The activation energy values of sweet potato starch were 173.85, 174.87, and 174.34 kJ·mol^−1^ based on Kissinger, Flynn–Wall–Ozawa, and Šatava–Šesták models, respectively. The activation energy values allow the development of a simplified approach to generally understand the thermal behavior of sweet potato starch in relation to food processing. The gaseous products from the pyrolysis of sweet potato starch were water, carbon dioxide, carbon monoxide, methane, and so on. The relationship between activation energy and conversion rate and the mechanism of degradation of sweet potato starch will be discussed in the future.

## Figures and Tables

**Table 1:** Parameters of sweet potato starch from TG curves.

Heating Rate	T_sd_/°C	T_5%_/°C	T_10%_/°C	T_50%_/°C	T_max_/°C
5 °C·min^−1^	45.50	83.07	289.95	316.98	308.37
10 °C·min^−1^	45.50	286.45	302.84	327.81	321.70
15 °C·min^−1^	45.50	314.07	318.98	349.88	325.98
20 °C·min^−1^	45.50	303.49	313.68	333.11	320.32

T_i_ is the temperature when α = i and T_max_ is the temperature at the maximum weight loss rate. T_sd_ is the starting temperature of decomposition.

**Table 2:** The activation energies of sweet potato starch using Šatava–Šesták model.

β/(°C·min^−1^)	5	10	15	20
R^2^	0.9940	0.9920	0.9990	0.9980
Slope	−9.495	−9.710	−9.549	−9.553
E(kJ·mol^−1^)	172.85	176.77	173.84	173.91
